# A Review on the Value of Imaging in Differentiating between Large Vessel Vasculitis and Atherosclerosis

**DOI:** 10.3390/jpm11030236

**Published:** 2021-03-23

**Authors:** Pieter H. Nienhuis, Gijs D. van Praagh, Andor W. J. M. Glaudemans, Elisabeth Brouwer, Riemer H. J. A. Slart

**Affiliations:** 1 Department of Nuclear Medicine and Molecular Imaging, Medical Imaging Center, University of Groningen, University Medical Center Groningen, 9700 RB Groningen, The Netherlands; g.d.van.praagh@umcg.nl (G.D.v.P.); a.w.m.j.glaudemans@umcg.nl (A.W.J.M.G.); r.h.j.a.slart@umcg.nl (R.H.J.A.S.); 2 Department of Rheumatology and Clinical Immunology, University of Groningen, University Medical Center Groningen, 9700 RB Groningen, The Netherlands; e.brouwer@umcg.nl; 3 Department of Biomedical Photonic Imaging, Faculty of Science and Technology, University of Twente, 7500 AE Enschede, The Netherlands

**Keywords:** large vessel vasculitis, atherosclerosis, imaging, FDG-PET, radiological imaging

## Abstract

Imaging is becoming increasingly important for the diagnosis of large vessel vasculitis (LVV). Atherosclerosis may be difficult to distinguish from LVV on imaging as both are inflammatory conditions of the arterial wall. Differentiating atherosclerosis from LVV is important to enable optimal diagnosis, risk assessment, and tailored treatment at a patient level. This paper reviews the current evidence of ultrasound (US), 2-deoxy-2-[18F]fluoro-D-glucose positron emission tomography (FDG-PET), computed tomography (CT), and magnetic resonance imaging (MRI) to distinguish LVV from atherosclerosis. In this review, we identified a total of eight studies comparing LVV patients to atherosclerosis patients using imaging—four US studies, two FDG-PET studies, and two CT studies. The included studies mostly applied different methodologies and outcome parameters to investigate vessel wall inflammation. This review reports the currently available evidence and provides recommendations on further methodological standardization methods and future directions for research.

## 1. Introduction

Large vessel vasculitis (LVV) is an inflammatory condition of the blood vessel wall affecting large- and medium-sized arteries. This may cause obstruction, ischemia, or aneurysm formation, resulting in vascular events, such as vision loss, cerebrovascular accidents, or aortic rupture [1].

The two major variants of LVV are giant cell arteritis (GCA) and Takayasu arteritis (TA). GCA and TA differ mainly in age of onset—older than 50 years and younger than 40 years, respectively—and the affected arteries. The aorta and its major branches are often affected in both variants. In GCA however, third-order branches of the aorta in the head and neck region, such as the temporal artery, are also commonly involved [2].

An early and accurate diagnosis is vital to prevent complications in patients with LVV. However, the diagnosis is difficult as there are no disease-specific signs, symptoms, or laboratory tests that can definitively prove or reject the presence of GCA or TA [3,4]. The “gold standard” for diagnosing GCA, a temporal artery biopsy, has a high specificity but lower sensitivity, depending on the included patients [5,6].

Consequently, to improve diagnosis, the role of imaging in LVV has been emerging over the past decade. Current recommendations include imaging early upon clinical suspicion of LVV [7]. Imaging techniques used to investigate LVV include ultrasound (US), magnetic resonance imaging (MRI), computed tomography (CT), and 2-deoxy-2-[18F]fluoro-D-glucose positron emission tomography (FDG-PET).

Similar to LVV, atherosclerosis is an inflammatory condition of the blood vessel wall characterized by an accumulation of activated immune cells, such as macrophages. Changes in vessel wall morphology also develop, mostly on the intimal side. Atherosclerotic lesions are patchy and result in plaque formation and calcification. Therefore, when imaging LVV patients, it may be difficult to distinguish between vasculitis and atherosclerosis. Distinguishing atherosclerosis from vasculitis is vital because both diseases require different treatment methods, and a personalized medicine approach [8].

The most commonly used imaging modalities in LVV are US and FDG-PET/CT. Several studies using these modalities reported atherosclerosis as a potential mimic for LVV. For US, the mainstay of recognizing LVV is based on the presence of a “halo sign”—a hypoechoic ring around the vessel wall [9]. However, there is evidence that the halo sign is also present in other vasculopathies, such as atherosclerosis [10]. Differentiating may be further complicated by the fact that the accuracy of US in LVV highly depends on the skills of the examiner and the US system used [5]. FDG-PET/CT displays glycolytic activity in tissues such as inflammatory lesions. Therefore, it is widely used to assess inflammation of the middle and large systemic arteries. Elevated FDG uptake can also be noticed in atheromatous plaques, making atherosclerosis a known confounder in the diagnosis of LVV with FDG-PET [11,12]. Currently, there is no clear consensus on how to distinguish FDG uptake by a vasculitic lesion from an atheromatous plaque [13].

MRI is mainly used in the smaller arteries of the head and neck for investigation of inflammation by LVV [9]. Typical vasculitic lesions show concentric wall thickening and contrast uptake around the inflamed artery [14], according to expert opinion. Atherosclerosis does not show any contrast enhancement and has a visible eccentric appearance. However, to the best of our knowledge, there is still much unknown about differentiating these lesions on MRI and recommendations for vasculitis do not mention how to differentiate. CT (angiography) visualizes vessel wall thickening and luminal changes in inflamed arteries [15]. Calcified plaques can be detected well on CT due to their high attenuating structures. Quantification of calcifications for risk assessment is done frequently, e.g., with the Agatston score [16]. Imaging noncalcified or high-risk atheromatous plaques with CT is, however, more challenging and may thus be difficult to distinguish from vasculitis [17].

Optimal diagnosis, risk assessment, and tailored treatment on patient-level is necessary, and this starts with the ability to discriminate LVV and atherosclerosis.

This review aims to evaluate the current evidence of imaging techniques to distinguish LVV from atherosclerosis.

## 2. Materials and Methods

### 2.1. Research Questions

The main research question of this review is: Can imaging findings of US, FDG-PET, CT, and MRI differentiate between large vessel vasculitis and atherosclerosis?

### 2.2. Search Strategy

The inclusion of appropriate studies was based on a literature search performed in the MEDLINE, EMBASE, and Web of Science electronic databases. The search strings used for each database is shown in the Appendix A.

Article title, authors, year of publication, and abstract were exported from the electronic databases and subsequently imported into Mendeley reference manager. Duplicates were removed based on suggestions in Mendeley and manually verified.

Reviewers independently screened all abstracts in Rayyan based on inclusion criteria. In case of disagreement about the eligibility of abstracts, consensus was reached through discussion. The resulting eligible articles were reviewed in full-text. These articles were cross-checked for important references. When these cited studies met our inclusion criteria, they were also included.

### 2.3. Inclusion Criteria

The resulting criteria for article inclusion were as follows: (1) studies including LVV patients, defined as GCA or TA; (2) studies including atherosclerosis patients; (3) studies in which US, MRI, CT, FDG-PET, or a combination of those were performed; (4) studies using parameters of vascular inflammation; (5) original research articles; (6) studies written in English.

### 2.4. Exclusion Criteria

Exclusion criteria were: (1) studies including patients with other types of vasculitis, such as Kawasaki or Behçet disease; (2) case reports, conference papers, animal studies, or (systematic) reviews.

### 2.5. Extraction of Study Characteristics

Several data were extracted from the included full-text papers. Study characteristics regarding the main study, including year of publication, type of study (prospective or retrospective), primary aim, methods used, and primary outcome of the study, were collected.

The outcome parameters regarding vascular inflammation (including—but not limited to—vessel wall thickness, tissue enhancement, FDG uptake, or the presence of a halo sign) were collected for the LVV patient group and the atherosclerosis patient group.

## 3. Results

**Figure 1:**
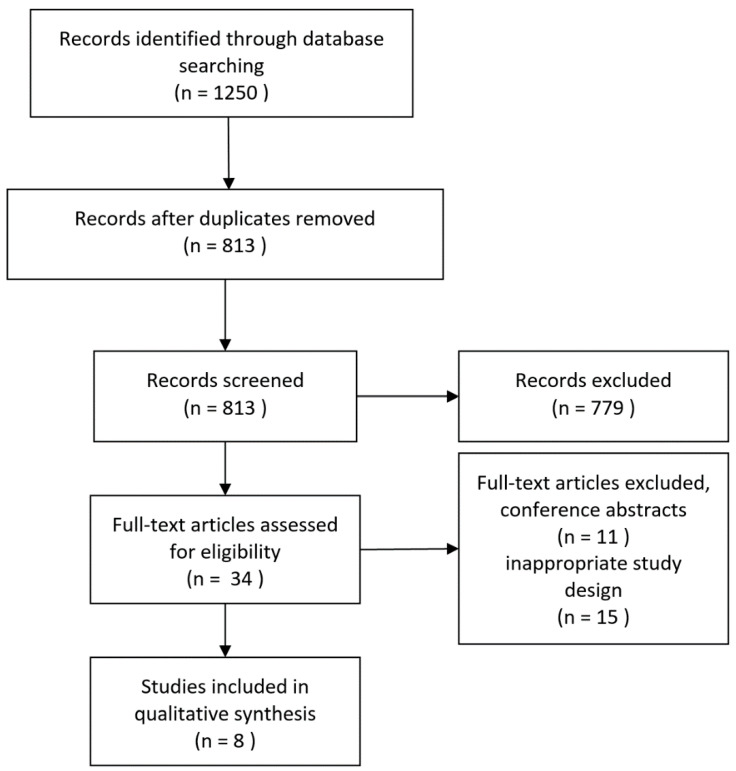
Flowchart of the literature review article selection process. Inappropriate study design refers to studies that did not include a well-defined LVV or atherosclerosis group or did not perform a comparative analysis between both.

An overview of the results of the literature search is shown in Figure 1. In total, eight original research articles met the inclusion criteria—a comparison of LVV patients and atherosclerosis patients imaged with US, CT, MRI, or FDG-PET—and, therefore, were included in this review.

Table 1 reveals the main characteristics of the included studies. Four studies were performed with US, two with FDG-PET, and two with CT. No studies with MRI were found.

In four studies, the primary objective was to assess the diagnostic accuracy of the imaging modality. Two studies included only TA patients, four studies included only GCA patients, and two studies included both. The included studies were heterogeneous in their study methods and did not use the same outcome parameters. Therefore, a direct comparison of the study results could not be performed, and only descriptive data are noted.

### 3.1. Ultrasound

Four studies contained data on US imaging in both LVV and atherosclerosis patients, see Table 2. Three studies considered a hypoechoic ring, or halo sign, as an outcome parameter to diagnose LVV. All three studies found that the presence of a halo sign was highly sensitive for GCA. However, two studies found that the halo sign can also be present in atherosclerosis. The study by Murgatroyd et al. found histologic evidence of moderate to severe atherosclerosis in patients with false-positive halo sign [18]. In a recent study, Fernàndez-Fernàndez et al. found that in a group of 305 patients with GCA-positive US examinations, 14 (4.6%) of patients were initially not diagnosed with GCA. Three out of these fourteen false-positive cases turned out to be atherosclerosis patients [19].

No other parameter of vascular inflammation was measured in the US studies. Karahaliou et al. demonstrated that blood flow abnormalities, mainly due to all degrees of stenosis, were present in both LVV and atherosclerosis and therefore not useful to differentiate between the diseases [20].

Tsai et al. compared carotid artery occlusion between atherosclerosis and TA patients. Homogeneous intima media thickening was considered a specific finding in TA patients, as opposed to heterogeneous thickening in atherosclerosis patients [21]. Moreover, they concluded that TA lesions were often more concentric (circumferential) compared with the eccentric (“off-center”) lesions in atherosclerosis. They also indicated the involvement of different locations of the diseases. Atherosclerosis had a predilection for occlusion of the carotid bifurcation, with 88% involvement in atherosclerosis patients compared with 27% in TA patients. Conversely, all included TA patients had some degree of stenosis of the subclavian artery compared with only 18% of atherosclerosis patients.

### 3.2. FDG-PET

Both studies on FDG-PET/CT imaging that were included used visual assessment methods as outcome parameters (Table 3). Grayson et al. included FDG-PET/CT assessment based on expert opinion (“gestalt”). Of all clinically active LVV (GCA and TA) patients, 85% was considered positive [22]. Using this method, 17% of patients in the hyperlipidemia group were falsely positive assigned as LVV on FDG-PET/CT.

A more standardized approach was taken in the study by Stellingwerff et al. by visually scoring FDG uptake in the vessels compared to the liver [23]. In grade 1, the vessel uptake was lower than the liver, in grade 2 equal to the liver, and in grade 3 higher than the liver. Considering grade 2 and 3 as positive for LVV, resulted in 100% sensitivity, considering only grade 3 as positive resulted in a 92% sensitivity. Importantly, sensitivity decreased for patients who were on treatment with glucocorticoids. Using the first threshold (similar to or higher than liver) resulted in diagnosing 63% of atherosclerosis patients as GCA. The latter threshold (higher than liver) was more specific for GCA with 21% of atherosclerosis cases being falsely positive for GCA.

Apart from uptake intensity, FDG uptake pattern can also be scored. A diffuse (homogeneous) FDG uptake pattern was present in all GCA patients, but only in 21% of atherosclerosis patients. When combining uptake intensity (uptake compared to liver) and a diffuse uptake pattern, GCA can be well differentiated from atherosclerosis with a 95% specificity. However, sensitivity for GCA decreased to 83%.

A semiquantitative approach to visual scoring was used in both studies. By counting the number of arteries and the intensity uptake grade, it is possible to better distinguish between LVV and (atherosclerotic) controls. Such a composite score, like the PETVAS score devised by Grayson et al., is likely to be higher in LVV compared to atherosclerosis, because of more intense FDG uptake in a higher number of arteries [22].

In the study by Stellingwerff et al., the maximal standardized uptake value (SUVmax) was also measured. This uptake parameter was, on average, higher in GCA patients compared to atherosclerosis patients but also showed overlap [23].

### 3.3. CT(A)

Two studies gathered data in vasculitis and atherosclerosis patients using CT Angiography, see Table 4. Both studies investigated the aorta. In 1995, Sharma et al. described vessel wall abnormalities in both abdominal and thoracic aorta, such as stenosis, dilatation, and wall thickening in patients with TA [24]. None of these vessel wall abnormalities was noticed in atherosclerosis patients. Calcification was present in all atherosclerosis patients and in 54% of TA patients.

Chowdhary et al. investigated patients with thoracic aortic aneurysms [25]. Patients with noninflammatory aneurysms more frequently had hyperlipidemia. These atherosclerotic patients showed increased aortic calcification compared to TA patients. This study also found aortic wall thickening of more than 3 mm in four atherosclerosis patients and in one LVV patient.

Additionally, the same study measured aortic diameters in the thoracic aorta. Aortic diameters differed in the aortic arch, where LVV patients had a slightly higher mean diameter than atherosclerosis patients.

## 4. Discussion

This review aimed to gather the currently available evidence for distinguishing LVV and atherosclerosis on imaging. Eight imaging studies were included based on their inclusion of separate groups of LVV and atherosclerosis patients. Several studies indicated that the most used diagnostic signs of LVV in US and FDG-PET, respectively, the halo sign and visual FDG uptake, may also be present in atherosclerosis patients. When visually scoring FDG uptake intensity and pattern, FDG-PET/CT attained 95% specificity for diagnosing LVV against an atherosclerotic control group. The two included CTA studies indicated calcification was more often seen in atherosclerosis patients. No MRI studies comparing LVV and atherosclerosis were found.

Three US studies indicated the presence of the halo sign in atherosclerosis patients. One study showed histologic evidence of atherosclerosis on temporal artery biopsy in patients with a positive halo sign [18]. However, these patients were clinically suspected of having GCA, and the temporal artery biopsy may have been a false negative. Two of these three articles were published 14 and 17 years ago. In more recent studies, the requirements for the halo sign were more standardized [26]. The included study from 2020 did show the presence of a halo sign using these standardized measures [19].

Another recent US study in atherosclerosis patients showed that thickening of the carotid artery walls correlated with thickening of the temporal artery walls, mimicking the halo sign [10]. As US is increasingly being used to diagnose LVV, there is a growing need to identify diseases that may mimic LVV diagnosis on US.

FDG-PET is already a proven method for measuring vascular inflammation in atherosclerotic plaques as well as in LVV [13,27]. However, only two studies identified in this review directly compared FDG-PET in atherosclerosis and LVV [22,23]. The presence of FDG uptake in atherosclerosis patients decreased the specificity of FDG-PET when diagnosing LVV. When used in conjunction with CT, an overlap of calcification and FDG uptake may be used to identify atherosclerosis [11]. However, FDG uptake is most prominent in the early stages of atherosclerosis and decreases when the plaque is calcified (stabilized non-vulnerable) [27,28]. Distinguishing between noncalcified atherosclerotic plaques and LVV may, thus, be extra challenging.

One way to discriminate between atherosclerosis and LVV is by objectivating the pattern of FDG uptake. One study included in this review showed that using a diffuse uptake pattern as a diagnostic criterium decreases the number of false positives, especially the number of false-positive atherosclerosis patients [23]. Atherosclerosis typically has more focal or “patchy” FDG uptake. Diffuse uptake as a diagnostic criterion has also been used in previous research [12], and recommended in the analysis of LVV [13,29]. In addition to uptake pattern, FDG uptake intensity is thought to be higher in LVV than in atherosclerosis [30]. This is reflected in the studies included in this review, where using a threshold for uptake higher than liver resulted in fewer false-positive atherosclerosis patients. Also, semiquantitative measurements such as SUVmax were higher in LVV patients. Careful interpretation of LVV patients with moderate FDG uptake is, therefore, important.

The location of the vessels may also differentiate between LVV and atherosclerosis, with atherosclerosis being more common in the abdominal aorta and iliofemoral arteries [30]. Cumulative FDG uptake scores—using the sum of uptake scores of multiple arteries—can help differentiate between LVV and atherosclerosis patients. However, such a cumulative score does not discriminate on the level of a single artery and, therefore, does not aid identifying atherosclerotic disease in LVV affected arteries.

A recent recommendation paper on FDG-PET/CT in LVV proposes further standardization of interpretation criteria and acknowledges the possible difficulties distinguishing atherosclerosis and LVV [13]. These include visually scoring uptake intensity compared to liver and noting its uptake pattern, with patchy uptake being more suggestive of atherosclerosis. The authors also recommended the use of a cumulative vascular FDG uptake score. Worldwide adoption of these standardized interpretation criteria will enable better comparison of LVV and atherosclerosis with FDG-PET/CT, expanding the current limited evidence on this subject.

The use of more semiquantitative measurements when interpreting FDG-PET/CT is mentioned by recommendation/position papers for both LVV and atherosclerosis [13,31]. Both papers mention the highest reproducibility when calculating the ratio of the standardized uptake values (SUV) of the vessel wall compared to SUV in a background organ. The use target-to-background ratio (TBR) may further increase standardization of FDG-PET/CT interpretation as well as provide an opportunity for monitoring vessel wall inflammation.

Two studies included in this review investigated CTA in TA patients. Apart from increased calcification in atherosclerosis patients, the results from these studies are less clear. One of the studies was published 25 years ago and only stated an absence of vessel wall abnormalities in atherosclerosis [24]. Additionally, the reference standard for the atherosclerosis group was not defined. The other CTA study compared aneurysm patients and found increased aortic vessel wall thickness in atherosclerosis and LVV patients. Importantly, this study also included 5 patients without LVV in the group of 17. Nonetheless, the study was included in this review because all patients in this group had aortitis.

Whereas calcified plaques are easily discernable on CT, detecting noncalcified, fatty plaques is more difficult [32]. Consequently, determining whether the cause of stenosis is atherosclerotic or vasculitic may also be challenging, especially in arteries that are commonly affected in both atherosclerosis and LVV, such as the carotid arteries. Distinguishing here may be especially important as severe complications such as transient ischemic attack and cerebrovascular accident can result from both atherosclerosis and LVV when the head/neck arteries are affected. CTA can be used to diagnose LVV based on morphological vessel wall abnormalities, showing concentric thickening stretching a long segment of an artery [33]. Atherosclerotic non-calcified plaques and vulnerability can also be assessed using CTA, showing eccentric and focal thickening of the arterial wall [34]. Although there is little evidence on the combined use of FDG-PET and CTA in LVV, its combined use may reliably diagnose LVV while also showing morphological changes to the vessel wall. This way, atherosclerosis may be distinguished reliably from LVV, also at the level of an individual artery.

No MRI studies met the inclusion criteria of this review. MRI is not commonly used in atherosclerosis imaging nor is it a first-line imaging technique in LVV [7]. Several MRI studies on intracranial vasculopathies did include vasculitis patients, although not identified as GCA or TA [14,35,36]. Schwarz et al. concluded MRI could be used well in differentiating between vasculitis and atherosclerosis, but the distinction in this study was mainly based on expert opinion. Vasculitis shows perivascular contrast enhancement and wall thickening can be characterized as concentric and in a long vessel segment. Conversely, atherosclerosis does not show any contrast enhancement, and wall thickening is eccentric and focal. The exact value of MRI in this matter should be further investigated.

The most frequently used imaging techniques in LVV, US and FDG-PET, may cause false-positive diagnoses in atherosclerosis patients. Discriminating atherosclerosis from vasculitis in LVV patients is vital to prevent unnecessary GC treatment in atherosclerotic patients. Moreover, there is evidence of accelerated atherosclerosis in LVV patients, in particular in TA [37]. GCA affects patients in the same age range where atherosclerosis is common, meaning they often overlap. Future studies will need to elucidate the extent to which atherosclerosis can mimic LVV and study the degree of atherosclerosis in LVV patients. Including atherosclerotic comparator groups in future LVV imaging studies is pivotal to addressing this question.

Additionally, studies using modern imaging systems are needed. High-resolution US, digital PET imaging, combined PET/MR, and previously mentioned combined PET/CTA may detect (noncalcified) underlying atheromatous plaques in LVV patients by visualizing the arterial wall in more detail [31]. Importantly, future imaging studies will need to include more quantified and standardized parameters with which these types of vascular inflammation can be differentiated. These parameters will subsequently allow us to recognize atherosclerotic plaques within LVV patients beyond using vascular calcification. Lastly, new identified parameters may allow the study of the interaction between the atherosclerotic and vasculitic process.

The reference standards used in the studies were also heterogeneous and 3 out of 8 studies were of retrospective design. Some studies defined those with an increased cardiovascular risk profile as atherosclerosis patients, based on clinical diagnosis or by hyperlipidemia. Other studies used histology or a plaque score. As there are no set criteria for diagnosing patients with atherosclerosis, multiple reference standards are possible. However, well defined and standardized methods are important to ensure reproducibility. Standardized cardiovascular risk profile scores and calcification scores may therefore be best suitable to define an atherosclerosis group. The latter may be achieved by using (low dose) CT scans to quantify the level of calcification, similar to the Agatston score [16]. Visual methods to assess the degree of calcification measurements may also be used, provided that CT is available [38]. Furthermore, artificial intelligence could be a valuable tool in differentiating between the (mixed) diseases. For example, neural networks have been proven to be strong in segmentation tasks and as classifiers [39]. One of the outcomes of the included studies of the current paper was that atherosclerotic uptake was more focal, while vasculitic uptake was more diffuse. Textural analysis of these neural networks and/or radiomics has the potential to prove this in more detail, including the location of the disease, as LVV is mainly in the adventitia and media of the arterial wall, and atherosclerosis at the intima, including the use of high resolution (digital) scanners to distinguish the vascular wall layers. In addition, it could vastly reduce analysis time of all vessels and potentially improve quantification of the diseases.

In general, there is a lack of common study methods and outcome parameters in the included studies, which prohibited us to perform a meta-analysis. However, this is, to the best of our knowledge, the first review to assess the studies in which atherosclerosis and LVV are compared on imaging. Hence, the little available evidence this review presents is important as a starting point for future research.

## 5. Conclusions

In conclusion, the evidence available in literature suggests that atherosclerosis can mimic imaging findings of LVV on US and FDG-PET. Hence, it may be difficult in some cases to differentiate between LVV and atherosclerosis, which lowers the diagnostic accuracy of these frequently used imaging methods. High intensity and diffuse uptake pattern on FDG-PET/CT showed the highest specificity distinguishing LVV from atherosclerosis. However, only few imaging studies directly compared atherosclerosis and LVV, and there is little standardization of study methods. Future, randomized, prospective study set-ups comparing atherosclerosis and LVV should be performed with standardized inclusion criteria and assessment methods.

## Figures and Tables

**Table 1:** Overview of the included studies and their aims and primary outcomes.

First Author	Year	Imaging Modality	Primary Aim	Primary Outcome
Sharma	1995	CT	Assess vessel wall changes in TA	TA patients show distinct changes in vessel wall morphology
Murgatroyd	2003	US	Evaluate the diagnostic accuracy of US in GCA	US shows a sensitivity 86% and a specificity of 68%
Tsai	2005	US	Identify the main cause of carotid artery occlusion	Atherosclerosis and TA are the two most common causes of carotid artery occlusion
Karahaliou	2006	US	Evaluate the diagnostic accuracy of US in GCA	US shows high sensitivity when bilateral halo sign is present
Chowdhary	2013	CT	Identify CT angiographic findings in aortitis	Idiopathic aortitis causes larger dilatation than noninflammatory aneurysms
Stellingwerff	2015	FDG-PET	To define optimal scoring methods for GCA	Visual scoring of vascular uptake compared to liver demonstrated the highest accuracy
Grayson	2018	FDG-PET	Assessing the role of FDG-PET as a biomarker in GCA	Higher FDG-PET scores resulted in a higher chance of relapse
Fernàndez-Fernàndez	2020	US	Frequency of US halo sign in non-GCA patients	There are other conditions than GCA that reveal the halo sign

CT = computed tomography; US = ultrasound; FDG-PET = 2-deoxy-2-[18F]fluoro-D-glucose positron emission tomography; TA = Takayasu Arteritis; GCA = Giant Cell Arteritis.

**Table 2:** Overview Outcome Parameters Ultrasound Studies.

First Author	Year	Study Design	Vasculitis Patients				Atherosclerosis Patients			Presence of Hypoechoic Ring (Halo Sign) Temporal Artery (%)		Blood Flow Abnormality (%)		Homogenous Echogenicity Carotid Artery (%)	
			Type of Vasculitis (GCA; TA)	Reference Diagnosis	Number of Patients	Mean Age	Reference Diagnosis	Number of Patients	Mean Age	Vasculitis Patients	Atherosclerosis Patients	Vasculitis Patients	Atherosclerosis Patients	Vasculitis Patients	Atherosclerosis Patients
Murgatroyd	2003	Prospective	GCA	Positive Temporal Artery Biopsy	7	-	Histology	8	-	6 (86)	6 (75)	-	-	-	-
Tsai	2005	Prospective	TA	Ishikawa Criteria	11	36	Clinical Diagnosis	17	70	-	-	-	-	0 (0)	11 (100)
Karahaliou	2006	Prospective	GCA	Clinical Diagnosis	22	70	Clinical Diagnosis of DM Type II or Stroke	15	73	18 (82)	0 (0)	9 (41)	6 (40)	-	-
Fernàndez-Fernàndez	2020	Retrospective	GCA	Clinical Diagnosis	291	-	3	-		291 * (100)	3 * (100)	-	-	-	-

* Patients included in this study were selected based on an US positive for GCA and, therefore, 100% of patients show the halo sign. GCA = Giant Cell Arteritis; TA = Takayasu Arteritis.

**Table 3:** Overview Outcome Parameters FDG-PET studies.

First Author	Year	Study Design	Vasculitis Patients				Atherosclerosis Patients			Number of Patients with Visual Uptake Similar to Liver (%); Higher than Liver (%)		Number of Patients with Diffuse Visual Uptake		Mean Number of Arteries with Increased Visual FDG Uptake (range)		Mean SUVmax in the Aorta (SD)		Number of Scans ** with Positive Visual ‘Gestalt’ LVV Assessment	
			Type of Vasculitis (GCA; TA)	Reference Diagnosis	Number of Patients	Mean Age	Reference Diagnosis	Number of Patients	Mean Age	Vasculitis Patients	Atherosclerosis Patients	Vasculitis Patients	Atherosclerosis Patients	Vasculitis Patients	Atherosclerosis Patients	Vasculitis Patients	Atherosclerosis Patients	Vasculitis Patients	Atherosclerosis Patients
Stellingwerff	2015	Retrospective	GCA	ACR Criteria; Positive TAB; Established Clinical Diagnosis	12	70	CT Calcified Plaque Score > 2	19	69	12 (100); 11 (92)	12 (63); 4 (21)	12 (100)	4 (21)	35 (19-40)	13 (5-27)	3.83 (1.10)	2.82 (0.76)	-	-
Grayson	2018	Prospective	GCA; TA *	ACR Criteria; Clinically Active Disease	25; 15 *	67; 44 *	Hyperlipidemia (>55 years and statin use)	35	64	-	-	-	-	22 (-); 19 * (-) ***	14 ***	-	-	34 (85)	6 (17)

* Data for the second patient group in this study. ** The study using this parameter used data for the number of scans, not numbers. *** The parameter in this study included two fewer arteries than the other study. LVV = Large vessel vasculitis; GCA = Giant Cell Arteritis; TA = Takayasu Arteritis.

**Table 4:** Overview Outcome Parameters CT(A) studies.

First Author	Year	Study Design	Vasculitis Patients				Atherosclerosis Patients			Patients with Aortic Stenosis or occlusion (%)		Patients with Aortic Dilative Lesions (%)		Patients with Aortic Wall Thickening (%)		Patients with Aortic Calcification (%)		Diameter Ascending Aorta mm (SD)		Diameter Aortic Arch mm (SD)		Diameter Descending Aorta mm (SD)	
			Type of Vasculitis (GCA; TA)	Reference Diagnosis	Number of Patients	Mean Age	Reference Diagnosis	Number of Patients	Mean Age	Vasculitis Patients	Atherosclerosis Patients	Vasculitis Patients	Atherosclerosis Patients	Vasculitis Patients	Atherosclerosis Patients	Vasculitis Patients	Atherosclerosis Patients	Vasculitis Patients	Atherosclerosis Patients	Vasculitis Patients	Atherosclerosis Patients	Vasculitis Patients	Atherosclerosis Patients
Sharma	1996	Prospective	TA	-	24	70	-	12	63	10 (42)	0 (0)	9 (38)	0 (0)	20 (83)	0 (0)	13 (54)	12 (100)	-	-	-	-	-	-
Chowdhary	2013	Retrospective	GCA ****	Clinical Diagnosis of Secondary Aortitis	16	36	Patients with noninflammatory aneurysms	18	70	-	-	-	-	1 (6)	4 (22)	1 (6)	10 (56)	53 (10)	49 (12)	35 (6)	31 (4)	36 (7)	33 (13)

**** This patient group included 10 GCA, 2 TA, 2 with bicuspid aortic valve, 1 seronegative arthritis, and 1 lupus patient. GCA = Giant cell arteritis; TA = Takayasu Arteritis.

## Data Availability

Not applicable.

## Appendix A

*Search string MEDLINE (search date: 01-12-2020, 424 results)*
(“Diagnostic Imaging”[Mesh] OR Diagnostic Ima*[tiab] OR Ultraso*[tiab] OR US[tiab] OR magnetic resonance [tiab] OR MRI[tiab] OR MRA[tiab] OR angiography[tiab] OR computed tomography[tiab] OR CT[tiab] OR CTA[tiab] OR positron emission tomography[tiab] OR PET[tiab])
AND
(“Giant Cell Arteritis”[Mesh] OR Vasculi*[tiab] OR LVV[tiab] OR large vessel vasculitis[tiab] OR giant cell arteritis[tiab] OR GCA[tiab] OR “Takayasu Arteritis”[Mesh] OR takayasu arteritis[tiab] OR TAK[tiab])
AND
(“Atherosclerosis”[Mesh] OR “Arteriosclerosis”[Mesh] OR athero*[tiab] OR vascular calcification[tiab])
NOT “Case Reports” [Publication Type]
*Search string EMBASE (search date: 01-12-2020, 519 results)*
((‘Diagnostic Ima*’ OR Ultraso* OR US OR ‘magnetic resonance’ OR MRI OR MRA OR angiography OR ‘computed tomography’ OR CT OR CTA OR ‘positron emission tomography’ OR PET):ab,ti)
AND
((Vasculi* OR LVV OR ‘large vessel vasculitis’ OR ‘giant cell arteritis’ OR GCA OR ‘takayasu arteritis’ OR TAK):ab,ti)
AND
((athero* OR ‘vascular calcification’):ab,ti)
NOT ‘case report’/exp
*Search string Web of Science, Core Collection (search date: 01-12-2020, 104 results)*
TS = (“Diagnostic Imaging” OR “Diagnostic Ima*’’ OR “Ultraso*’’ OR “US’’ OR “magnetic resonance’’ OR “MRI’’ OR “MRA’’ OR “angiography’’ OR “computed tomography’’ OR “CT’’ OR “CTA’’ OR “positron emission tomography’’ OR “PET’’)
AND
TI = (“Giant Cell Arteritis” OR ‘’Vasculi*’’ OR ‘’LVV’’ OR ‘’large vessel vasculitis’’ OR ‘’giant cell arteritis’’ OR ‘’GCA’’ OR “Takayasu Arteritis” OR ‘’TAK’’)
AND
TS = (“Atherosclerosis” OR “Arteriosclerosis” OR ‘’athero*’’ OR ‘’vascular calcification’’)
